# Identification and molecular characterization of a novel *Chlamydomonas reinhardtii *mutant defective in chlorophyll biosynthesis

**DOI:** 10.12688/f1000research.2-138.v2

**Published:** 2013-07-29

**Authors:** Phillip B Grovenstein, Darryel A Wilson, Cameron G Lennox, Katherine P Smith, Alisha A Contractor, Jonathan L Mincey, Kathryn D Lankford, Jacqueline M Smith, Tashana C Haye, Mautusi Mitra

**Affiliations:** 1 Department of Biology, University of West Georgia, Carrollton GA, 30118, USA

## Abstract

The green micro-alga *Chlamydomonas* *reinhardtii* is an elegant model organism to study all aspects of oxygenic photosynthesis. Chlorophyll (Chl) and heme are major tetrapyrroles that play an essential role in energy metabolism in photosynthetic organisms and are synthesized via a common branched tetrapyrrole biosynthetic pathway. One of the enzymes in the pathway is Mg chelatase (MgChel) which inserts Mg ^2+^ into protoporphyrin IX (PPIX, proto) to form magnesium-protoporphyrin IX (MgPPIX, Mgproto), the first biosynthetic intermediate in the Chl branch. MgChel is a multimeric enzyme that consists of three subunits designated CHLD, CHLI and CHLH. Plants have two isozymes of CHLI (CHLI1 and CHLI2) which are 70%-81% identical in protein sequences. Although the functional role of CHLI1 is well characterized, that of CHLI2 is not. We have isolated a non-photosynthetic light sensitive mutant *5A7* by random DNA insertional mutagenesis that is devoid of any detectable Chl. PCR based analyses show that *5A7* is missing the *CHLI1* gene and at least eight additional functionally uncharacterized genes. *5A7* has an intact *CHLI2* gene. Complementation with a functional copy of the *CHLI1 *gene restored Chl biosynthesis, photo-autotrophic growth and light tolerance in *5A7*. We have identified the first *chli1 (chli1-1)* mutant of *Chlamydomonas reinhardtii *and in green algae. Our results show that in the wild type *Chlamydomonas* CHLI2 protein amount is lower than that of CHLI1 and the *chli1-1* mutant has a drastic reduction in CHLI2 protein levels although it possesses the *CHLI2* gene. Our *chli1-1 *mutant opens up new avenues to explore the functional roles of CHLI1 and CHLI2 in Chl biosynthesis in *Chlamydomonas*, which has never been studied before.

## Introduction

The green micro-alga *Chlamydomonas reinhardtii* possesses a photosynthetic apparatus very similar to that of higher plants, can grow photo-autotrophically and heterotrophically (it can metabolize exogenous acetate as a carbon source) and possesses a completely sequenced genome ^1^. These attributes make it an elegant model organism to study oxygenic photosynthesis and chloroplast biogenesis ^2, 3^. In photosynthetic organisms, tetrapyrroles like Chl and heme are essential for energy metabolism (i.e. photosynthesis and respiration). Biosynthesis of Chl and heme occur via a common branched pathway that involves both nuclear- and chloroplast-encoded enzymes in most photosynthetic organisms ^4^. In photosynthetic eukaryotes, 5-aminolevulinic acid (ALA) is synthesized from glutamine through glutamyl-tRNA ^5^. Conversion of ALA through several steps yields protoporphyrin IX (PPIX), the last common precursor for both heme and Chl biosynthesis ^5^. Ferrochelatase inserts iron in the center of PPIX thus committing it to the heme branch of the pathway. Insertion of Mg ^2+^ in PPIX by MgChel leads to Mgproto, the first biosynthetic intermediate in the Chl branch ^6^. Magnesium chelatase has three subunits, which are CHLD, CHLH and CHLI ^7^. The ATP-dependent catalytic mechanism of the heterotrimeric MgChel complex includes at least two steps ^7– 9^: an activation step, followed by the Mg ^2+^ insertion ^8^. Activation of MgChel with ATP involves CHLD and CHLI while CHLH is required for the chelation step ^10^. CHLI belongs to the AAA+ family of ATPases. Plants have two isozymes of CHLI1 (CHLI1 and CHLI2) which are 70%–81% identical in protein sequences ^10^. Although the functional role of CHLI1 is well characterized, that of CHLI2 is not. Most of the data on CHLI comes from studies on *Arabidopsis thaliana chli* mutants and the functional significance of CHLI1 and CHLI2 has not been studied in green algae ^10– 16^. In *Arabidopsis CHLI2* plays a limited role in Chl biosynthesis because of its lower expression level compared to that of CHLI1 ^12– 15^. In *Arabidopsis* the CHLI2 protein amount is lower than that of CHLI1. When overexpressed, CHLI2 can fully rescue an *Arabidopsis chli1chli2* double mutant ^12^.

We have isolated the first ( *chli1-1*) mutant of *Chlamydomonas reinhardtii* ( *5A7*) which possesses an intact *CHLI2* gene. Transformation of *5A7* with a functional copy of the *CHLI1* gene restored Chl biosynthesis. Western analyses show that the CHLI2 protein level is lower than that of CHLI1 in the wild type strain and CHLI2 protein is barely detectable in the mutant strain. In this study, we present our molecular data on the identification of the mutation locus in *5A7* and its complementation.

## Materials and methods

### Algal media and cultures

*Chlamydomonas* strains 4A+ (a gift from Dr. Krishna Niyogi (UC, Berkeley), *5A7/chli1-1* (generated by our laboratory) and *chli1-1* rescued transformants (generated by our laboratory) were grown either in Tris-Acetate Phosphate (TAP) heterotrophic media or in Sueoka’s High Salt (HS) photo-autotrophic media. TAP and HS liquid media and agar plates were prepared in the lab using reagents from Fisher Scientific (Pittsburg, PA) according to the protocol given in Gorman and Levine (1965) ^17^ and Sueoka (1960) ^18^, respectively. The 4A+ strain and *chli1-1* rescued transformants were maintained on TAP agar plates and TAP+zeocin (Sigma, St. Louis, MO) plates, respectively under dim light intensities (10–15 µmol photons m ^-2^s ^-1^) at 25°C. The final zeocin concentration was 15 µg/ml). The *chli1-1* mutant ( *5A7*) was maintained in the dark on TAP 1.5% agar plates containing 10 µg/ml of paromomycin (Sigma, St. Louis, MO). Liquid algal cultures used for RNA and genomic DNA extractions and protein analyses were grown in 100 ml flasks on the New Brunswick Scientific Excella E5 platform shaker (Enfield, CT) at 150 rpm in the dark or in the dim light.

### Generation of the *5A7* mutant

**Figure 1.**
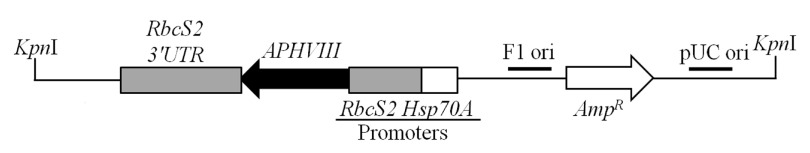
Linearized pBC1 plasmid used for random insertional mutagenesis. The cleavage site of the *Kpn*1 restriction enzyme, used for linearization of the vector is shown. *APHVIII* is under the control of combo promoters consisting of the promoter of the gene encoding the small subunit of Rubisco (RbcS2) and the promoter of the gene encoding the heat shock protein 70A (Hsp70A). pBC1 is a phagemid and its F1 origin (F1 ori) and pUC origin (pUC ori) are shown. The size of the plasmid is 4763 bp.

The purified pBC1 plasmid from the DH5α *Escherichia coli* clone harboring the pBC1 plasmid (obtained from Dr. Krishna Niyogi’s laboratory at UC, Berkeley) was used for random DNA insertional mutagenesis. This plasmid contains two antibiotic resistance genes: *APHVIII* and *Amp ^R^* ( Figure 1). *APHVIII* confers resistance against the antibiotic paromomycin (Sigma, St. Louis MO) and was used as a selection marker for screening of *Chlamydomonas* transformants. *Amp ^R^* was used as a selection marker for screening of *E. coli* clones harboring the pBC1 plasmid. *E. coli* was grown in 1 l of Luria Bertani (LB) broth containing 1% tryptone, 0.5% of yeast extract, 1% NaCl and ampicillin [final concentration of ampicillin:100 µg/ml]. LB reagent was prepared in the laboratory using reagents purchased from Fisher (Pittsburgh, PA). Ampicillin was purchased from Fisher (Pittsburgh, PA). The culture was incubated at 37°C overnight. Plasmid purification from *E. coli* cells was facilitated by Qiagen plasmid mega kit according to the protocol given in the technical manual (Qiagen, Valencia, CA). Once purified from *E. coli*, the circular pBC1 vector was linearized with the restriction enzyme *Kpn*1 (NEB, Beverly, MA) according to the protocol given in the technical manual. The linearized DNA was purified using a QIAEX II gel extraction kit (Qiagen, Valencia, CA) according to the protocol given in the technical manual. All agarose DNA gel electrophoresis was visualized by BioRad Molecular Imager Gel Doc XR+ (BioRad, Hercules, CA). Transformation of parental strain 4A+ by the linearized pBC1 vector was performed utilizing the glass bead transformation technique described by Kindle *et al.* (1989) ^19^ and Dent *et al.* (2005) ^2^. Transformants were plated onto fresh TAP agar plates containing 10 µg/ml paromomycin (TAP+P) in the dark. Single colonies of mutants were picked and transferred onto fresh TAP+P plates using a numbered grid layout. Screening of photosynthetic and pigment deficient mutants was done by visual inspection and monitoring of growth under different light intensities in heterotrophic, mixotrophic and photo-autotrophic conditions ^2^.

### Genomic DNA and RNA extraction

4A+, *chli1-1* rescued transformants complements and *5A7/chli1-1* were grown in TAP liquid media in the dark to a cell density of about 5 × 10 ^6^ cells/ml of the culture. Genomic DNA was purified using a phenol-chloroform extraction method ^19^. RNA extraction was facilitated by TRIzol reagent from Invitrogen (Carlsbad, CA) following the protocol in the technical manual. DNA and RNA concentrations were measured using a Nanodrop 1000 spectrophotometer from Thermo Fisher Scientific (Wilmington, DE). DNase treatment was performed using Ambion’s TURBO DNA-free kit from Invitrogen (Carlsbad, CA) following the protocol in the technical manual to remove genomic DNA from the RNA preparation. Generation of cDNA was performed using Life Technologies Superscript III First-Strand Synthesis System from Invitrogen (Carlsbad, CA) following the protocol in the technical manual.

### Thermal asymmetric interLaced PCR

TAIL (Thermal Asymmetric InterLaced) PCR was implemented, following the protocol of Dent *et al.* (2005) ^2^. This protocol was implemented with one modification of utilizing a non-degenerate primer (AD2) derived from the original degenerate primer (AD) for TAIL PCR as this non-degenerate primer was giving us optimum yield without generating excess nonspecific products. HotStar Taq Plus DNA polymerase kit reagents (Qiagen, Valencia, CA) were used for PCR. The PCR reaction mixture consisted of 1× PCR buffer, 200 µM of each dNTP, 1× Q-solution, 2.5 units of HotStar Taq Plus DNA polymerase, 60 pmoles of the non-degenerate primer AD2 and 5 pmol of the *APHVIII* specific primer. Primers were ordered from IDT (Skokie, IL; Table 1). The non-degenerate primer AD2 has a T *_m_* of 46°C while the three *APHVIII* specific primers used had T *_m_* ranging from 58°C to 64°C. PCR cycling programs were created using the program given in Dent *et al.* (2005) ^2^. TAIL1 PCR product was diluted 10 and 25-fold and 2 μl of the diluted TAIL1 PCR product was used for TAIL2 PCR reactions. The TAIL2 PCR product was gel purified using QIAEX II gel extraction kit (Qiagen, Valencia, CA) according to the protocol given in the technical manual. The purified TAIL2 PCR product was sequenced at the UC, Berkeley DNA Sequencing Facility (Berkeley, CA).

**Table 1.** List of primers used for Thermal Asymmetric InterLaced (TAIL) PCR, verification of TAIL-PCR product and DNA sequencing. These primers were used to generate the data in Figure 3 and Figure 4.

Primer name	Sequence of primer	Location
AD	5´-NTC GWG WTS CNA GC-3´	Random degenerate primer
AD2	5´-ATCGTGTTCCCAGC-3´	Non-degenerate primer derived from the primer AD
1F	5´-AAA GAC TGA TCA GCA CGA AAC GGG-3´	*APHVIII* 3´UTR
2F	5´-TAA GCT ACC GCT TCA GCA CTT GAG-3´	*APHVIII* 3´UTR
2R	5´-CTC AAG TGC TGA AGC GGT AGC TTA-3´	*APHVIII* 3´UTR
3R	5´-TCT TCT GAG GGA CCT GAT GGT GTT-3´	*APHVIII* 3´UTR
4R	5´-GGG CGG TAT CGG AGG AAA AGC TG-3´	*APHVIII* 3´UTR

### Genomic and reverse transcription PCR

Primers were designed based on genomic DNA sequences available in the *Chlamydomonas* genome database in Phytozome. Amplifications of genomic DNA and cDNA were executed using MJ Research PTC-200 Peltier Thermal Cycler (Watertown, MA). HotStar Taq Plus DNA polymerase kit (Qiagen, Valencia, CA) was used for PCR following the cycling conditions given in the Qiagen protocol booklet. Annealing temperature was between 55 and 60°C depending on the T *_m_* of the primers. Extension time was varied according to the size of the PCR product amplified. Final extension was set at 72°C for ten minutes. All genomic and reverse transcription PCR products were amplified for a total of thirty-five cycles. 50–150 ng of genomic DNA or cDNA templates were used for PCR reactions. For semi-quantitative RT-PCR using *CHLI1* and *CHLI2* gene specific primers, 3 μg of total RNA was converted into cDNA and then 150 ng of cDNA templates were used for RT-PCR. Sequences of primers used for genomic and RT-PCR are shown in Table 2, Table 3 and Table 4.

**Table 2.** List of primers used for amplifying *CHLI1* and four neighboring genes downstream of *CHLI1*. These primers were used to generate the data in Figure 6A. The gene loci in Phytozome ( http://www.phytozome.net/) are: *CHLI1* (Cre06.g306300), *UP1* (Cre06.g306250), *UP2* (Cre06.g306200), *UP3* (Cre06.g306150) and *UP4* (Cre06.g306100).

Primer name	Sequence of primer	Gene
CHLI1AF	5´-ACTACGACTTCCGCGTCAAGATCA-3´	*CHLI1*
CHLI1BR	5´-CATGCCGAACACCTGCTTGAAGAT-3´	*CHLI1*
PB138	5´-ATGACCGTAACGCTGCGTAC-3´	*UP1*
PB139	5´-CGTGAACGACAGTGTTTAGCG-3´	*UP1*
PB132	5´-AAGGGCATCAGCTACAAGGTC-3´	*UP2*
PB133	5´-GGCATCGAGGATGTATTGGTTG-3´	*UP2*
UP3F	5´-GGC ACA CAA GCG TGA TTT TCT GG-3´	*UP3*
UP3R	5´-CGG CAC GTC GAA GAC AAA CT-3´	*UP3*
UP4F	5´-CTT TGA CCT GCA AAG AGA GAA AGC G-3´	*UP4*
UP4R	5´-CAC CAC CTT GAT GCC CTT GAG-3´	*UP4*

**Table 3.** List of primers used for amplifying *CHLI1*, four neighboring genes upstream of *CHLI1* and the actin gene and transcript. These primers were used to generate the data in Figure 6B and 6C. The gene loci in Phytozome ( http://www.phytozome.net/) are: *CHLI1* (Cre06.g306300), *FDX3* (Cre06.g306350), *AMT* (g7098), *UP5* (Cre06.g306450) and *UP6* (Cre06.g306500) and *Actin* (Cre13.g603700).

Primer name	Sequence of primer	Gene
CHLI1AF	5´-ACTACGACTTCCGCGTCAAGATCA-3´	*CHLI1*
CHLI1BR	5´-CATGCCGAACACCTGCTTGAAGAT-3´	*CHLI1*
PB134	5´-CTGGAGCGCACCTTTATGAAG-3´	*AMT*
PB135	5´-AGTGGAACAGGTTCTCGATGAC-3´	*AMT*
PB132	5´-AAGGGCATCAGCTACAAGGTC-3´	*FDX3*
PB133	5´-GGCATCGAGGATGTATTGGTTG-3´	*FDX3*
UP5F	5´-GGG CAA CTG GAG CTT TGG C-3´	*UP5*
UP5R	5´-CGT CTA TGT GCG CCA CGT C-3´	*UP5*
UP6F	5´-GCA ACT GGA GCT TCG GCG-3´	*UP6*
UP6R	5´-CGT AGG CGC CAA ACA CCG-3´	*UP6*
F2	5´-ACGACACCACCTTCAACTCCATCA-3´	*Actin*
R2	5´-TTAGAAGCACTTCCGGTGCACGAT-3´	*Actin*

**Table 4.** List of primers for amplifying *CHLI* transcripts and complement testing. These primers were used in the experiments that generated the data in Figure 7 and Figure 10 and also used for *CHLI1* cDNA amplification for cloning.

Primer name	Sequence of primer	Gene/purpose
CHLI1CR	5´-TTGACCCTTTGACACGAACCAACC-3´	*CHLI1*
CHLI1BR	5´-CATGCCGAACACCTGCTTGAAGAT-3´	*CHLI1*
CHLI1AF	5´-ACTACGACTTCCGCGTCAAGATCA-3´	*CHLI1*
CHLI2BF	5´-TGACGCATTTGTGGACTCGTGCAA-3´	*CHLI2*
CHLI2CR	5´-CACACTTACACGTTCACGCAGCAA-3´	*CHLI2*
CHLI1XF	5´-GGAATTCCATATGGCCTGAACATGCGTGTTTC-3´	*CHLI1* cDNA amplification for cloning
CHLI1XR	5´-CCGGAATTCTTACTCCATGCCGAACACCTGCTT-3´	*CHLI1* cDNA amplification for cloning
PsaDF1	5´-CCACTGCTACTCACAACAAGCCCA-3´	Complementation testing

### Cloning of the *CHLI1* cDNA in the pDBle vector

The pDBle vector (obtained from Dr. Saul Purton, University College London, UK) was double-digested with restriction enzymes *Eco*R1 and *Nde*1 (NEB, Beverly, MA) according to the protocol given in the technical manual. The *CHLI1* cDNA template was amplified using primers given in Table 4. Ligation of the double digested ( *Nde*I and *Eco*R1 digested) *CHLI1* cDNA and the *Nde*I/ *Eco*RI double-digested pDBle vector was done using the T4 ligase and 1 mM ATP (NEB, Beverly, MA). Chemically competent (CaCl _2_ treated) *E. coli* cells were used for transformation. After transformation, *E. coli* cells were plated on LB+ampicillin (100 µg/ml) plates and incubated at 37°C overnight. Single colonies were picked the next day and plasmids were isolated from these clones. Isolated plasmids were double-digested with *Eco*R1 and *Nde*1 to verify the cloning of the *CHLI1* cDNA. The *CHLI1-pDBle* construct from the selected clone was sequenced by the UC, Berkeley DNA Sequencing Facility (Berkeley, CA). Chromas Lite ( http://technelysium.com.au/) and BLAST were used to analyze DNA sequences.

### Generation and screening of *chli1-1* rescued transformants

Complementation of the *chli1-1* was performed utilizing the glass bead transformation technique described by Kindle *et al.* 1989 ^20^. 2 µg of the linearized *CHLI1-pDBle* was used to complement *chli1-1*. Transformed cells were plated onto fresh TAP plates containing 15 µg/ml zeocin (Z) and placed in the dark at 25°C. Single colonies were picked and transferred onto fresh TAP+Z plates using a numbered grid template for screening of potential *chli1-1* rescued transformants. Screening of *chli1-1* rescued transformants was done by visual inspection of green coloration and monitoring growth of light adapted complement strain cells either on TAP in the dark or in the dim light or HS plates under medium light (300 μmol photons m ^-2^s ^-1^).

### Cellular protein analysis

*Chlamydomonas* cells from different strains grown in TAP in the dark were harvested, washed twice with fresh medium and resuspended in TEN buffer (10 mM Tris-HCl, 10 mM EDTA and 150 mM NaCl; pH 8). Protein concentrations of samples were determined by the method of Lowry *et al.* (1951) ^21^ with bovine serum albumin as standard. Gel lanes were either loaded with an equal amount of Chl (4 μg Chl) or with 40 μg of protein. Resuspended cell suspension was mixed in a 1:1 ratio with the sample solubilization buffer SDS-urea buffer (150 mM Tris-HCl, pH 6.8; 7% w/v SDS; 10% w/v glycerol; 2 M urea, bromophenol blue and 10% β-mercaptoethanol) and were incubated at room temperature for about thirty minutes, with intermittent vortexing. The sample solubilization buffer was prepared according to the protocol of Smith *et al.* (1990) ^22^ using reagents from Fisher (Pittsburgh, PA). After incubation, the solubilized protein samples were vortexed and spun at a maximum speed of 20,000 *g* in a microcentrifuge for five minutes at 4°C. The soluble fraction was loaded on a "any kD ^™^ Mini-PROTEAN ^®^ TGX ^™^ Precast Gel" (BioRad, Hercules, CA) and SDS-PAGE analysis was performed according to Laemmli (1970) ^23^ using a Page Ruler prestained or unstained protein ladder (Fermentas, Glen Burnie, Maryland) at a constant current of 80 V for 2 hours. Gels were stained with colloidal Coomassie Gel code blue stain reagent (Thermo Fisher Scientific, Rockford, IL) for protein visualization.

### Western analysis

Electrophoretic transfer of the SDS-PAGE resolved proteins onto an Immobilon P–PVDF membrane (Millipore, Billerica, MA) was carried out for 2 hours at a constant current of 400 mA in the transfer buffer (25 mM Tris, 192 mM glycine and 20% methanol). The CHLI1 polyclonal antibody was raised in rabbit against the full length *Arabidopsis thaliana* CHLI1 mature protein that lacks the predicted transit peptide ^24^. This antibody is a gift from Dr. Robert Larkin (Michigan State University). CHLI1 primary antibodies were diluted to a ratio of 1:2,000 before being used as a primary probe. The secondary antibodies used for Western blotting were conjugated to horseradish peroxidase (Pierce protein research product, Thermo Fisher Scientific, Rockford, IL) and diluted to a ratio of 1:20,000 with the antibody buffer. Western blots were developed by using the Supersignal West Pico chemiluminescent substrate kit (Pierce protein research product, Thermo Fisher Scientific, Rockford, IL).

### Cell counts and chlorophyll extraction

Cell density (number of cells per ml of the culture) was calculated by counting the cells using a Neubauer ultraplane hemacytometer (Hausser Scientific, Horsham, PA). Pigments from intact cells were extracted in 80% acetone and cell debris was removed by centrifugation at 10,000 *g* for 5 minutes. The absorbance of the supernatant was measured with a Beckman Coulter DU 730 Life science UV/Vis spectrophotometer (Brea, CA). Chl *a* and *b* concentrations were determined by Arnon (1949) ^25^ equations, with corrections as described by Melis *et al.* (1987) ^26^.

## Results

### Generation and identification of the mutant *5A7*

**Figure 2.**
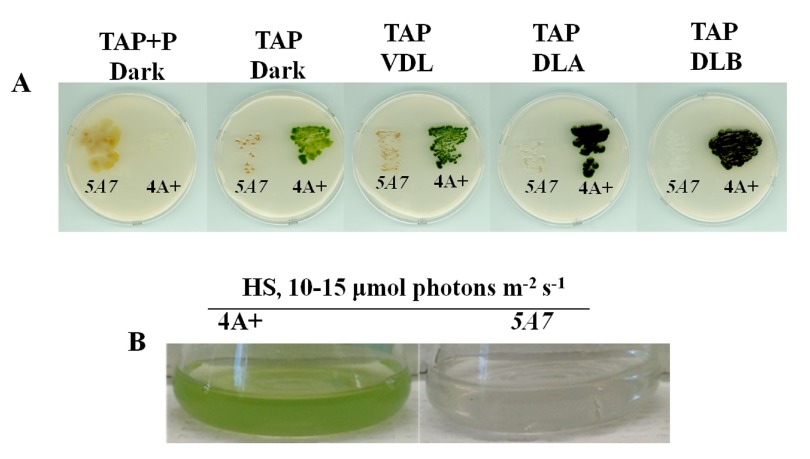
Growth phenotype of *5A7*. ( **A**) This figure shows the phenotypic difference of *5A7* compared to the parental strain, 4A+ on heterotrophic/mixotrophic agar media (TAP) plates under five different light conditions: dark + paromomycin (P), dark, very dim light (VDL, 2–4 μmol photons m ^-2^s ^-1^), dim light A (DLA, 10–15 μmol photons m ^-2^s ^-1^) and dim light B (DLB, 20–25 μmol photons m ^-2^s ^-1^). ( **B**) This figure shows the growth phenotype of *5A7* in liquid photo-autotrophic media (HS) under dim light (DL = 10–15 μmol photons m ^-2^s ^-1^).

Mutant *5A7* was generated by random insertional mutagenesis of the *Chlamydomonas reinhardtii* wild type strain 4A+ (137c genetic background). *5A7* lacks detectable chlorophyll, appears yellowish-brown in color and grows only under heterotrophic conditions in the dark or in the dim light in the presence of acetate in the growth media ( Figure 2). It is incapable of photosynthesis and is sensitive to light intensities higher than 20 μmol photons m ^-2^s ^-1^ ( Figure 2A).

### Molecular characterization of the mutation in *5A7*

**Figure 3.**
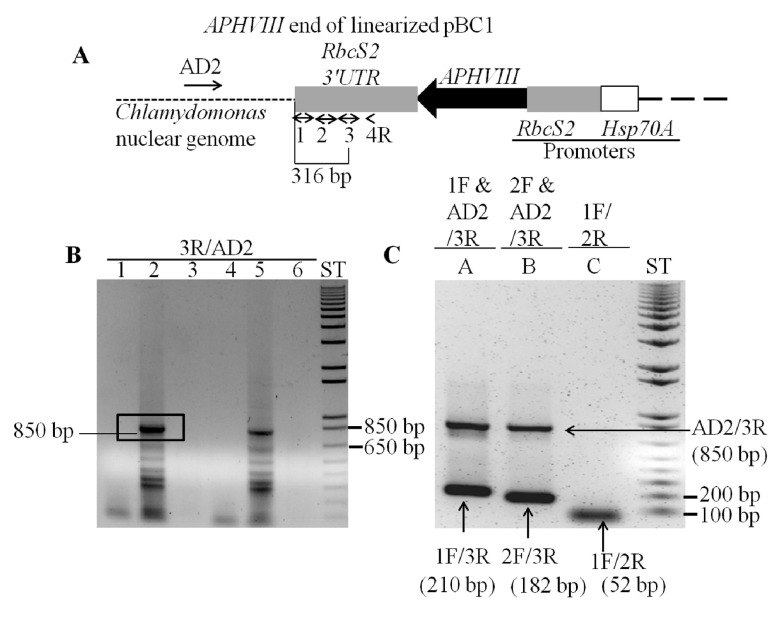
Locating the *APHVIII* flanking genomic sequence in *5A7*. ( **A**) A diagram showing a truncated pBC1 illustrating the *APHVIII* end of the linearized pBC1 vector. Primers used for PCR are shown by numbered black arrows. Thermal Asymmetric InterLaced 1 (TAIL1) PCR was performed using primer 4R and AD2. ( **B**) TAIL2 PCR was performed using primer 3R and AD2. Lanes 1 and 4 are zero DNA lanes; in lane 2, a 10-fold diluted TAIL1 PCR product was used for TAIL2 PCR; in lane 5, a 25-fold diluted TAIL1 PCR product was used for TAIL2 PCR; lanes 3 and 6 are blank lanes. The 850 bp product used for DNA sequencing is highlighted. ( **C**) Gel purified DNA product (850 bp) from the TAIL2 PCR was used to verify if the product was specific to the *APHVIII* gene. F and R stand for forward and reverse primers, respectively. AD2 is a non-degenerate primer. PCR primer names are labeled on the top of the gel. In lanes A and B, where triple primers were used for PCR, PCR products are labeled by the corresponding primer combinations that gave rise to the specific product. PCR product sizes are shown beside the primer combinations. All primer sequences are shown in Table 1. ST stands for 1 kb plus ladder (Invitrogen, Carlsbad, CA). DNA samples were run on a 1% agarose gel.

**Figure 4.**
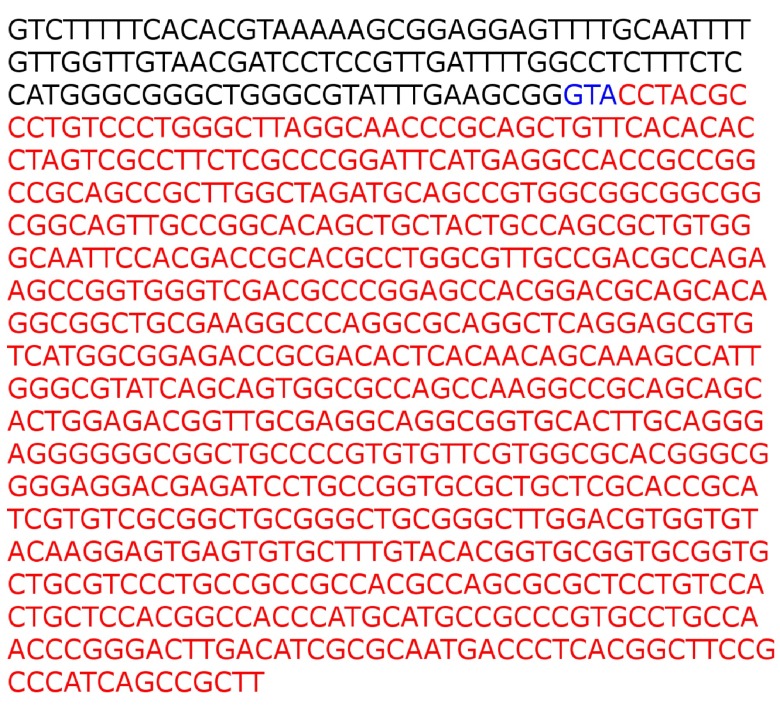
The *APHVIII* flanking genomic DNA sequence in *5A7*. Primer 2R ( Table 1), specific to the 3´UTR of the *APHVIII* gene was used for sequencing the 850 bp Thermal Asymmetric InterLaced 2 (TAIL2) PCR product. 3´UTR sequence of *APHVIII* is in bold black, extra nucleotide additions are in bold blue. The flanking *Chlamydomonas UP6* genomic sequence is denoted in red. The *APHVIII* end of the plasmid has been inserted after the eighth nucleotide in the fourth exon of *UP6* gene.

The linearized plasmid pBC1 was used to generate *5A7* ( Figure 1). To find the insertion of the *APHVIII* end of the plasmid in *5A7*, a modified TAIL (Thermal Asymmetric InterLaced) PCR method was used. Figure 3A shows the position of the vector specific TAIL PCR primers and also shows the arbitrary position of the random non-degenerate primer. A 850 bp DNA product from TAIL2 PCR was purified from the agarose gel ( Figure 3B, Table 1). This purified DNA product was used for PCR using internal primers specific to the 3´UTR (UnTranslated Region) of the *APHVIII* gene. The PCR results confirmed that the 850 bp DNA product contains the 3´UTR of the *APHVIII* gene ( Figure 3C). Sequencing of the 850 bp TAIL2 PCR product revealed that the *APHVIII* end of the plasmid has been inserted in the fourth exon of a hypothetical gene which we have named as *UP6* ( Figure 4). *UP6* (Cre06.g306500) is located on chromosome 6.

**Figure 5.**
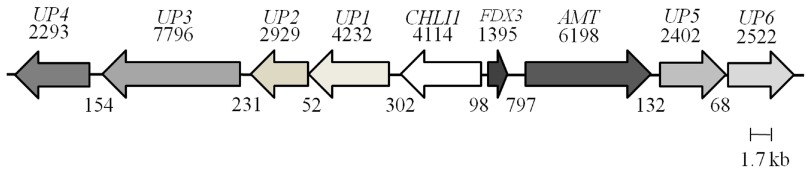
A schematic map of the *UP6* locus on chromosome 6. The map shows a 35.7 kb genomic DNA region that harbors the *UP6* and eight genes located upstream of it. Each arrow represents a gene. The name of the gene is given on top of the arrow. The black numbers on the top of arrows denote sizes of genes (bp) while black numbers below denote distances in between genes (bp).

**Figure 6.**
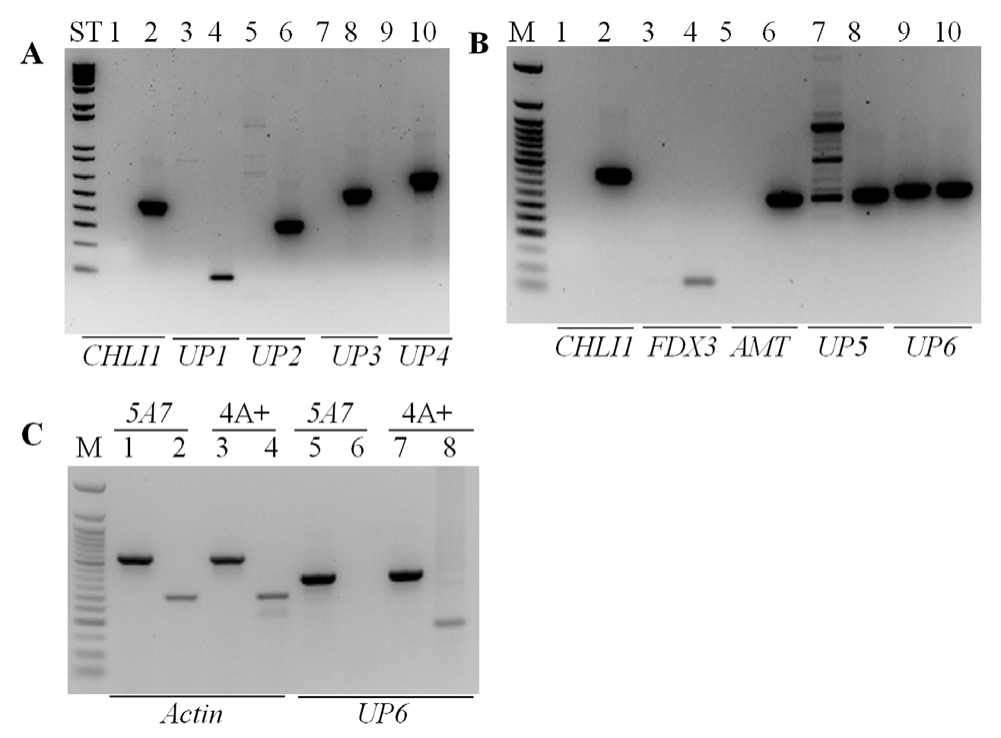
PCR analyses using primers specific to eight genes neighboring the *CHLI1* locus. ( **A**) PCR using the genomic DNA of *5A7* and 4A+ with primers specific to *CHLI1* and four neighboring genes ( *UP1*, *UP2*, *UP3*, *UP4*) downstream of the *CHLI1* gene. The sizes of the genomic DNA PCR products for *CHLI1*, *UP1*, *UP2*, *UP3* and *UP4* are, 459, 100, 342, 550 and 672 (bp), respectively. Odd numbered lanes denote *5A7*; even numbered lanes denote 4A+; ST denotes 1 kb plus DNA ladder. ( **B**) PCR using the genomic DNA of *5A7* and 4A+ with primers specific to *CHLI1* and four neighboring genes ( *FDX3, AMT, UP5* and *UP6*) upstream of the *CHLI1* gene. The sizes of the genomic DNA PCR products for *CHLI1*, *FDX3, AMT, UP5* and *UP6* are, 459, 90, 369, 379 and 369 (bp), respectively. Odd numbered lanes denote *5A7*; even numbered lanes denote 4A+; M denotes 50 bp DNA ladder (NEB, Beverly, MA). ( **C**) PCR and RT-PCR with *UP6* gene specific primers using the *5A7* and 4A+ genomic DNA and cDNA. Actin was used as a control. Actin genomic and cDNA product sizes are 527 and 305 (bp), respectively. Odd numbered lanes denote genomic DNA PCR products; even numbered lanes denote cDNA products. All primers used spanned an intron. M denotes 50 bp DNA ladder. All DNA samples were run on a 1.8% agarose gel. Gene names are given at the bottom of the gel. Primer sequences are shown in Table 2 and Table 3.

Figure 5 shows a schematic map of the *UP6* locus with its eight neighboring genes *UP4* (Cre06.g306100), *UP3* (Cre06.g306150), *UP1* (Cre06.g306250), *UP2* (Cre06.g306200) *CHLI1* (Cre06.g306300), *FDX3* (Cre06.g306350), *AMT* (g7098) and *UP5* (Cre06.g306450). It is to be noted that we have named all of these genes arbitrarily for our study except for the *CHLI1* and *FDX3* genes, which were annotated in the *Chlamydomonas* genome database. Readers are requested to identify these unknown genes by the gene locus number (Cre or g number) in the Phytozome database. PCR analyses with the genomic DNA of 4A+ and *5A7* were performed using primers specific to four neighboring genes upstream of the *CHLI1* (including *UP6*) and four neighboring genes downstream of the *CHLI1* locus ( Table 2 and Table 3; Figure 6A and 6B). PCR analyses revealed that all eight genes neighboring the *CHLI1* locus were deleted or displaced from their native location ( Figure 6A and 6B). *UP5* primers gave nonspecific multiple products in *5A7* ( Figure 6B). The first two exons of *UP6* are present in the *5A7* genome as the *UP6* primers spanning the first and the second exon, gave similar genomic DNA PCR product of the expected size as in the 4A+ lane ( Figure 6B). Reverse transcription (RT-PCR) analyses using the same *UP6* primers on *5A7* and 4A+ cDNA did not yield a PCR product in *5A7* unlike that in 4A+ ( Figure 6C; Table 3). This shows that the insertion of the plasmid in the fourth exon of *UP6* in *5A7* has hampered the transcription of the *UP6* gene.

Taken together the data shows that at least a 35,715 bp genomic region has been deleted and or/displaced when the plasmid got inserted in the *5A7* genome. Except for the *CHLI1* gene, the functions of the remaining eight genes (including *UP6*) are not known. We do not yet know the exact location of the pUC origin (pUC ori) end of the plasmid ( Figure 1) in the *5A7* genome.

### Checking for the absence/presence of the *CHLI1* transcript and the *CHLI2* gene and transcript

**Figure 7.**
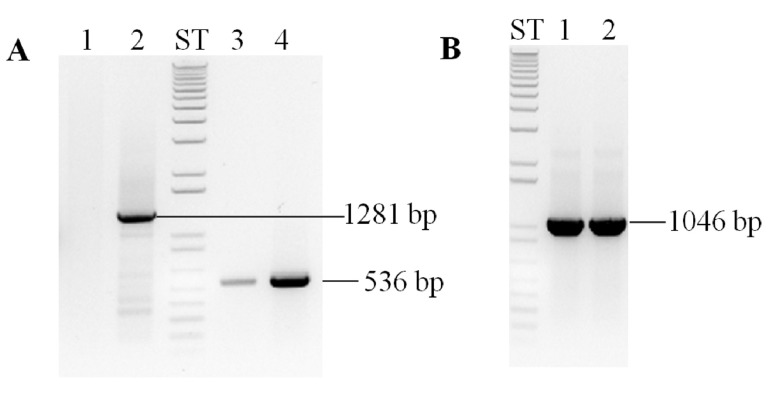
PCR analyses to check for the presence of the *CHLI1* and *CHLI2*. ( **A**) Semi-quantitative RT-PCR analyses of *5A7* and 4A+ using *CHLI1* and *CHLI2* primers. ( **B**) PCR analyses using *5A7* and 4A+ genomic DNA with *CHLI2* gene specific primers. Odd numbered lanes denote *5A7*; even numbered lanes denote 4A+. PCR product sizes (bp) are labeled. ST denotes 1 kb plus DNA ladder. All DNA samples were run on a 1.8% agarose gel. Gene names are given at the top of the gel. Primer sequences are shown in Table 4.

As CHLI plays a role in Chl biosynthesis, we checked for the presence/absence of the *CHLI1* and *CHLI2* in *5A7*. RT-PCR results show that *CHLI1* transcript is absent and *CHLI2* transcript is present in *5A7* ( Figure 7A, Table 4). Figure 7B shows the presence of the *CHLI2* gene in *5A7*.

### Complementation of *5A7*

**Figure 8.**
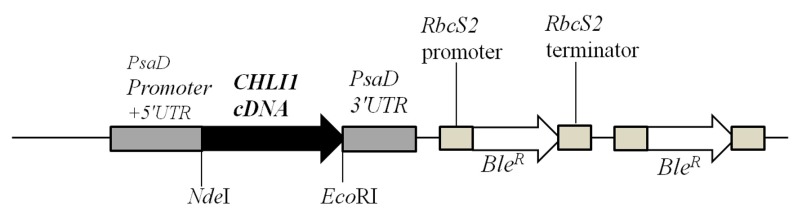
A schematic figure of the pDBle vector used for complementation of *chli1-1*. *Nde*I/ *Eco*R1 double digested *CHLI1* cDNA (1260 bp) was cloned into the *Nde*I/ *Eco*RI double digested pDBle plasmid. Primers used for amplification of *CHLI1* cDNA are shown in Table 4. *CHLI1* expression is driven by the constitutive *PsaD* promoter. *Nde*I and *Eco*RI restriction sites are labeled. pDBle contains two copies of *Ble ^R^* genes driven by the Rubisco ( *RbcS2*) promoter. The size of the *CHLI1*- *pDBle* construct is 7957 bp. Black arrow and white arrow denotes *CHLI1* cDNA and *Ble ^R^* gene, respectively. Grey boxes denote UnTranslated regions (UTR).

**Figure 9.**
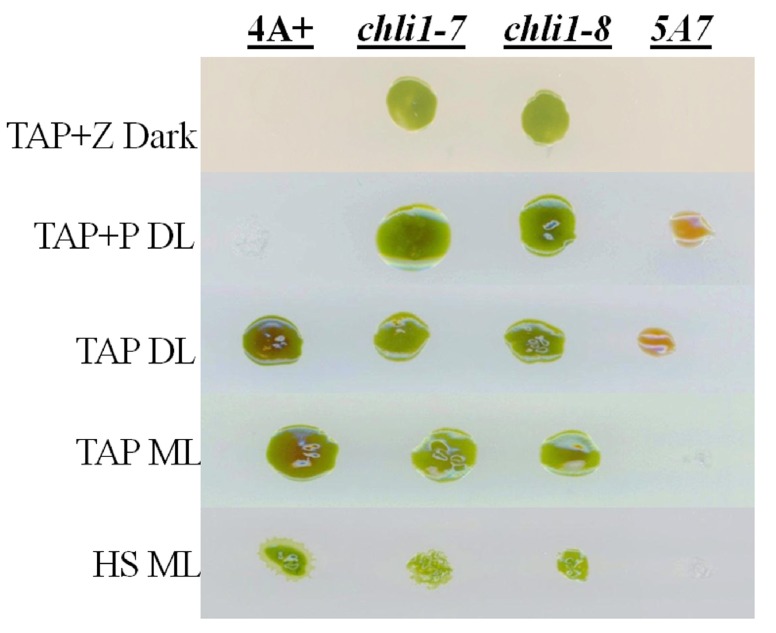
Growth phenotype analysis of *chli1-1* rescued transformants. *chli1-1* rescued transformants, *chli1-7* and *chli1-8*, were grown with *5A7/chli1-1* and 4A+ under five growth conditions: TAP+Z (zeocin) in the dark, TAP in dim light (DL) (15 µmol photons m ^-2^s ^-1^), TAP+P (paromomycin) in DL, TAP in medium light ML (300 µmol photons m ^-2^s ^-1^) and HS in ML.

We will be referring to strain *5A7* as *chli1-1* from here onward. As our *chli1-1* lacks Chl and CHLI1 is involved in Chl biosynthesis, we cloned the *CHLI1* cDNA in the pDBle vector to transform *chli1-1* ( Figure 8, Table 4). *CHLI1* expression is driven by the constitutive *PsaD* promoter in the *CHLI1-pDBle* construct ( Figure 8). pDBle has two *Ble* genes that confer resistance to the antibiotic zeocin. Figure 9 shows growth phenotypes of two *chli1-1* rescued transformants ( *chli1-7* and *chli1-8*); *chli1-1* and 4A+. *chli1-1* rescued transformants are able to synthesize Chl, are not light sensitive and are capable of photosynthesis ( Figure 9). As the *chli1-1* rescued transformants harbor the *Ble* gene (from the pDBle vector) and *APHVIII* gene (derived from the parental strain *chli1-1*), they can grow both on zeocin and paromomycin media plates unlike *chli1-1* and 4A+ ( Figure 9).

**Figure 10.**
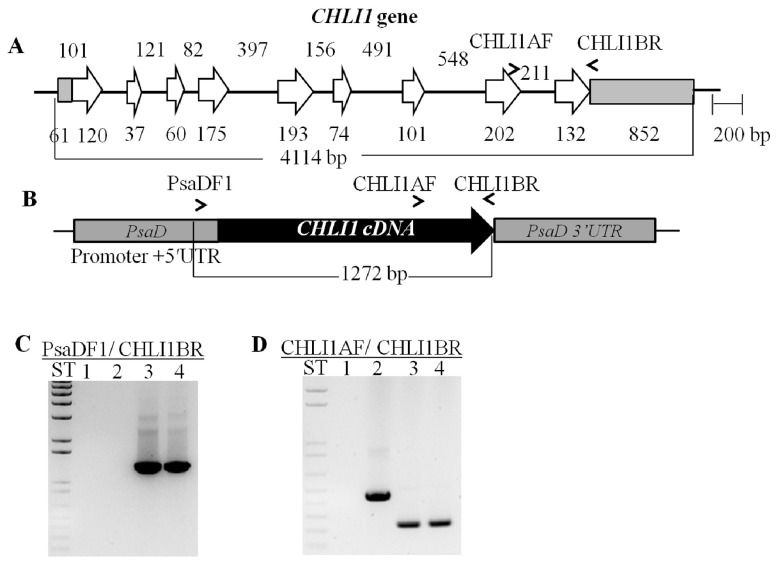
Molecular analysis of *chli1-1* rescued transformants. ( **A**) A schematic of the native *CHLI1* gene. The tan bars denote UnTranslated Regions (UTRs), the white arrows represent exons and the black lines denote introns. ( **B**) A schematic of the *CHLI1-pDBle* complementation vector containing the *CHLI1* cDNA. *PsaD* promoter, 5´UTR, 3´UTR and *CHLI1* specific primers are labeled. ( **C**) Genomic DNA PCR using a *PsaD* 5´UTR specific primer and a *CHLI1* specific primer. Product size: 1272 bp. Lane 1: *chli1-1*; Lane 2: 4A+; Lane 3: *chli1-7*; Lane 4: *chli1-8*. ST represents 1 kb plus DNA ladder. ( **D**) Genomic DNA PCR using *CHLI1* specific primers. Genomic DNA product size: 459 bp; cDNA product size: 249 bp. Lanes 3 and 4 show smaller PCR products compared to that in lane 2 as cDNA was used for complementation. Lane 1: *chli1-1*; Lane 2: 4A+; Lane 3: *chli1-7*; Lane 4: *chli1-8*. ST represents 1 kb plus DNA ladder. All primer sequences are shown in Table 4.

**Figure 11.**
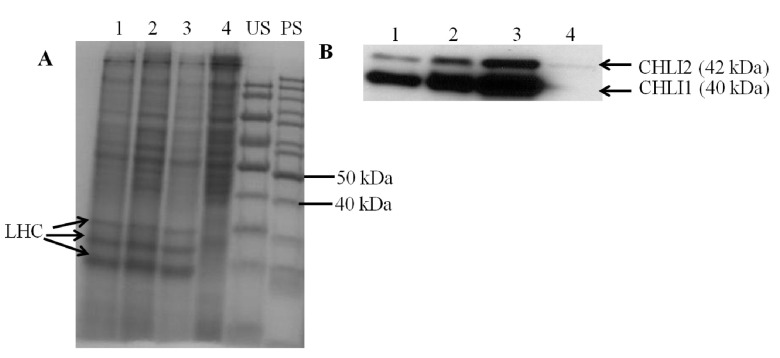
SDS-PAGE and Western analyses. ( **A**) A stained protein gel. Lanes 1, 2, 3 and 4 represent *chli1-8*, *chli1-7*, 4A+ and *chli1-1*, respectively. Light harvesting complex (LHC) protein bands are labeled. PS and US denote pre stained and unstained molecular weight protein ladders, respectively. Total cell extract of different strains were loaded on equal Chl basis (4 µg of Chl) in lanes 1, 2 and 3. In lane 4, 40 µg of protein (the maximum amount of protein that can be loaded on a mini protein gel) was loaded as *chli1-1* lacks Chl. ( **B**) Western analyses using a CHLI1 antibody generated against the *Arabidopsis* CHLI1 protein. Lanes 1, 2, 3 and 4 represent *chli1-8*, *chli1-7*, 4A+ and *chli1-1*, respectively. CHLI1 (40 kDa) and CHLI2 (42 kDa) proteins detected by the antibody are labeled.

Chl analyses show that both *chli1-1* rescued transformants are about 33–46% Chl deficient. *chli1-1* rescued transformants have a similar Chl *a*/ *b* ratio as that of the wild type ( Table 5, Data File below). Figure 10A and 10B show a schematic figure of the native *Chlamydomonas CHLI1* gene and the trans *CHLI1* gene used for complementation, respectively. PCR analyses using the genomic DNA show that the *chli1-1* rescued transformants have the trans *CHLI1* gene ( Figure 10C and 10D). In Figure 10D the genomic DNA PCR product sizes in the two *chli1-1* rescued transformant lanes are smaller than that in the 4A+ lane as we have cloned the *CHLI1* cDNA for complementation. The *Chlamydomonas* CHLI1 protein has about 71% sequence identity to the *Arabidopsis* CHLI1 protein. Figure 11A shows a stained protein gel. The two *chli1-1* rescued transformants and the 4A+ were loaded on an equal Chl basis in each lane in the protein gel ( Figure 11A). As *chli1-1* lacks Chl, the maximum amount of protein (40 µg) that can be loaded in a mini protein gel, was used ( Figure 11A). Light harvesting complex proteins (LHCs) can barely be detected in the *chli1-1* mutant ( Figure 11A).

**Table 5.** Spectrophotometric analyses of chlorophyll in 4A+, *chli1-1*, *chli1-7* and *chli1-8* strains. Chlorophyll analyses were done on three biological replicates for each strain. Strains were grown mixotrophically in TAP under 15–20 µmol photons m ^-2^s ^-1^. Mean values are shown in the table. Statistical error (± SD) was ≤10% of the values shown. ND: not detected.

Parameter	4A+	Strains *chli1-1*	*chli1-7*	*chli1-8*
Chl/cell (nmoles/cell)	3.6 × 10 ^-6^	ND	2.0 × 10 ^-6^	2.5 × 10 ^-6^
Chl *a/b* ratio	2.8	–	2.7	2.8

Spectrophotometric analyses of chlorophyll in 4A+, chli1, chli1-7 and chli1-8 strains of Chlamydomonas reinhardtii Chlorophyll analyses were done on three biological replicates for each strain. Strains were grown mixotrophically in TAP medium under 15-20 µmol photons m-2s-1. Chlorophyl measurements are in nmol/ml; column I is the uncorrected value, column J is the corrected value. Measurements were taken on 07/02/2013. Chl - chlorophyll Click here for additional data file. Copyright: © 2013 Grovenstein PB et al. 2013 Data associated with the article are available under the terms of the Creative Commons Zero "No rights reserved" data waiver (CC0 1.0 Public domain dedication).

Western analyses of the two *chli1-1* rescued transformants with a CHLI1 antibody show that the CHLI1 protein is absent in the *chli1-1* mutant but present in the *chli1-1* rescued transformants ( Figure 11B). Western analyses also show that the *Arabidopsis* CHLI1 antibody detects both the CHLI1 (40 kDa) and CHLI2 (42 kDa) protein in *Chlamydomonas* as the *Chlamydomonas* CHLI2 has about 62% sequence identity to the *Arabidopsis* CHLI1 ( Figure 11B). In the wild type the CHLI2 protein amount is much lower than that of CHLI1. As the *chli1-1* rescued transformants are Chl deficient compared to the wild type, the two rescued transformant lanes show higher amount of protein loadings ( Figure 11A). Although more protein was loaded in the *chli1-1* lane in the protein gel compared to that in the 4A+ and the *chli1-1* rescued transformant lanes, the CHLI2 protein was barely detectable in *5A7* ( Figure 11B).

## Discussion

*5A7* is the first *chli1-1* mutant to be identified in *C. reinhardtii* and in green algae. *CHLI1* deletion has affected Chl biosynthesis and photosynthetic growth in the *chli1-1* mutant ( Figure 2). Over-accumulation of photo-excitable PPIX leads to photo-oxidative damage to the cells in presence of light and oxygen ^4– 6^. The light sensitivity of the *chli1-1* is most probably due to an over-accumulation of PPIX which occurs due to the inactivity of MgChel enzyme which converts PPIX to MgPPIX. Future HPLC (High Performance Liquid Chromatography) analyses of steady state tetrapyrrole intermediates will confirm this hypothesis.

Based on the current molecular analyses, our *chli1-1* mutant has a deletion of at least nine genes (including the *CHLI1* gene). Currently we are investigating the exact insertion point of the pUC ori end of the plasmid in the *chli1-1* genome ( Figure 1). This will provide us with a precise estimate of the number of gene deletions in *chli1-1*. Although complementation of *chli1-1* with the *CHLI1* gene restored Chl biosynthesis, tolerance to high light levels and photo-autotrophic growth, *chli1-1* rescued transformants are still Chl deficient to some extent ( Table 5). This is probably due to a lower expression of the CHLI1 protein in these *chli1-1* rescued transformants ( Figure 11). Semi-quantitative RT-PCR shows that the CHLI2 transcript level in *chli1-1* is much lower than that in the wild type strain ( Figure 7). Western analyses show that the CHLI2 protein level is severely reduced in the *chli1-1* mutant ( Figure 11). Real Time PCR analyses can be used to confirm whether the reduction in the CHLI2 protein level is due to a low abundance of the *CHLI2* transcript. Additionally, the roles of any of the other missing eight genes in Chl biosynthesis cannot be ruled out as currently the functions of these genes are unknown.

In *Arabidopsis*, it has been shown that CHLI2 does play a limited role in Chl biosynthesis in the absence of CHLI1 ^12– 15^. *chli1-1* possesses an intact *CHLI2* gene but the *CHLI2* protein is barely detectable in the mutant. This raises two questions:

1) Is the low abundance of the CHLI2 protein a general effect or is it a specific effect of the *CHLI1* mutation?

2) Is the total absence of Chl in strain *chli1-1* due to the specific absence of CHLI1 or due to the absence of both CHLI1 and the near absence of CHLI2 protein?

The first question can be addressed by performing Western analyses of *chli1-1* with antibodies raised against any non-photosynthetic and/or photosynthetic protein. If the low abundance of the CHLI2 protein is due to a general effect of the mutation, there will be an overall reduction of different cellular proteins. The second question can be addressed by overexpressing CHLI2 in *chli1-1* to see if Chl biosynthesis can occur in the absence of CHLI1 or by silencing CHLI2 in the wild type strain using RNA interference or micro RNA based techniques.

Norflurazon (NF) causes photo-oxidative damage to the chloroplast by inhibiting carotenoid biosynthesis. ^24, 27– 31^. In *Arabidopsis* MgPPIX is hypothesized to be a retrograde signal from the chloroplast to the nucleus on the basis of data obtained with mutants that are defective in the NF, induced down-regulation of the transcription of the light harvesting complex protein B(LHCB) expression [gun (genomes uncoupled) phenotype] ^27, 28^. In *Arabidopsis*, there are controversies regarding whether *chli1* mutants are *gun* mutants ^12, 29– 31^. To date in *Arabidopsis*, MgPPIX mediated regulation of genes encoding only photosynthetic or chloroplastic proteins, have been documented ^27– 31^. In *Chlamydomonas*, hemin and MgPPIX has been shown to induce global changes in nuclear gene expression in *Chlamydomonas*, unlike that in *Arabidopsis* ^27– 28, 32– 37^. In *Chlamydomonas*, the above mentioned tetrapyrroles altered expressions of genes encoding TCA cycle enzymes, heme binding proteins and stress response proteins as well as proteins involved in protein folding and degradation (eg. heat shock proteins) ^34– 37^. Hence the roles of tetrapyrroles in retrograde signaling appears to be distinct in green algae and in higher plants. In summary, in the future our *chli1-1* mutant can be used to clarify the functional role of CHLI1 and CHLI2 in Chl biosynthesis in *C. reinhardtii*.
