# Supplementary material for: Identification and molecular characterization of a novel Chlamydomonas reinhardtii mutant defective in chlorophyll biosynthesis
Source: F1000Res. 2013 Jul 29;2:138. Originally published 2013 Jun 10. [Version 2] doi: 10.12688/f1000research.2-138.v2 (PMC3901506; doi:10.12688/f1000research.2-138.v2)
Supplement: Peer review history

# Reviewer report: Reviewer response for version 2

**DOI:** 10.5256/f1000research.1956.r1504

**Published:** 2021-09-16

**Authors:** Ruby A. Ynalvez

**Affiliations:** 1 Department of Biology and Chemistry, Texas A&M International University, Laredo, TX, USA

I no longer have any comment nor suggestion. The authors have addressed all my queries and have incorporated my suggested recommendations.

Reviewer Expertise:

NA

I confirm that I have read this submission and believe that I have an appropriate level of expertise to confirm that it is of an acceptable scientific standard.

# Reviewer report: Reviewer response for version 2

**DOI:** 10.5256/f1000research.1956.r1651

**Published:** 2013-08-30

**Authors:** David Oppenheimer

**Affiliations:** 1 Department of Botany, University of Florida, Gainesville, FL, USA

In this version of the manuscript, the authors have made all of the suggested revisions that I requested. I have no further requests from the authors.

Reviewer Expertise:

NA

I confirm that I have read this submission and believe that I have an appropriate level of expertise to confirm that it is of an acceptable scientific standard.

# Reviewer report: Reviewer response for version 2

**DOI:** 10.5256/f1000research.1956.r1587

**Published:** 2013-08-28

**Authors:** Tatsuru Masuda

**Affiliations:** 1 Environmental Sciences, University of Tokyo, Tokyo, Japan

I have reviewed the manuscript by Grovenstein *et al.* In this manuscript, the authors identified a Chl deficient mutant of *Chlamydomonas reinhardtii* by insertional mutagenesis. 

As described in the manuscript, *Arabidopsis* possesses two CHLI isoforms, CHLI1 and CHLI2, like *Chlamydomonas.* However, it is not clear whether these isoforms are paralogous or not among these two photosynthetic organisms. The authors should perform phylogenetic analysis to show whether these isoforms are truly phylogenetically related or not.

Considering the *chli1-1 *mutant is Chl deficient, it is reasonable to assume that CHLI2 is not functional in *Chlamydomonas*. In my understanding, the expression of *CHLI2 *is not affected by the mutation of *CHLI1* gene in *Arabidopsis.* The upper band of Fig.11B could be a non-specific band of CHLI1 protein that hides the original band of CHLI2 in Western blot analysis. In this sense, the expression analysis of *CHLI2,* as well as functional complementation with *CHLI2* in the *chli1-1 *mutant, are apparently necessary for further understanding.

Specific comments:

In Fig. 7, the authors performed semi-quantitative RT-PCR analysis of *CHLI1 *and *CHLI2*. As the authors discussed quantities of these transcripts in the Discussion section (p.10), it is necessary to show the levels of housekeeping genes such as *ACTIN8 *or *Ubiquitin10 *as loading control.

Reviewer Expertise:

NA

I confirm that I have read this submission and believe that I have an appropriate level of expertise to confirm that it is of an acceptable scientific standard.

# Reviewer report: Reviewer response for version 2

**DOI:** 10.5256/f1000research.1956.r1254

**Published:** 2013-08-13

**Authors:** Ruby A. Ynalvez

**Affiliations:** 1 Department of Biology and Chemistry, Texas A&M International University, Laredo, TX, USA

The authors did incorporate the suggestions from version 1 into version 2 of the article.  On my end,  version 2 is already approved with no more revisions required.

Reviewer Expertise:

NA

I confirm that I have read this submission and believe that I have an appropriate level of expertise to confirm that it is of an acceptable scientific standard.

# Reviewer report: Reviewer response for version 1

**DOI:** 10.5256/f1000research.1581.r1027

**Published:** 2013-06-26

**Authors:** Ruby A. Ynalvez

**Affiliations:** 1 Department of Biology and Chemistry, Texas A&M International University, Laredo, TX, USA

The title is appropriate for the content of the article. In addition, the abstract represents a suitable summary of this interesting work. The authors were able to successfully isolate a novel *Chlamydomonas reinhardtii *mutant defective in chlorophyll biosynthesis. Using molecular biology techniques they have provided sufficient evidence that the novel mutant is missing a CHLI1 gene and having an intact CHLI2 gene. Their results also provided evidence that CHLI1 in *C. reinhardtii* is important in chlorophyll biosynthesis and that the presence of its homolog CHLI2 is not sufficient to make up for the function of CHLI1 in chlorophyll biosynthesis. The growth phenotype analysis of the rescued transformants has shown clearly the role of CHLI1 in chlorophyll biosynthesis. It would be interesting to know what percentage of rescued transformants was in their complementation analysis.   Overall this is an interesting article. More importantly, this work reports the first *chli1 *mutant to be identified in *C. reinhardtii, *as well as in green algae. The *chli1 *mutant will open up new avenues to further explore the functional roles of CHLI1 and CHLI2 in chlorophyll biosynthesis in particular in *C. reinhardtii*.

**Suggested revisions:**

On page 6, sentence 2 “ *5A7 *lacks detectable chlorophyll, appears yellowish-brown in color and grows only under heterotrophic conditions in the dark or in the dim light in the presence of acetate in the growth media (Figure 1).” *Figure 1 should be Figure 2. On page 6, sentence 3 “It is incapable of photosynthesis and is sensitive to light intensities higher than 20 μmol photons m-2s-1 (Figure 2),” ---*can be more clearly referred to as Figure 2A instead of Figure 2 only.   On page 8, Figures 5A, 5B and 5C mentioned in text actually refers to Figures 6A, 6B and 6C.

Reviewer Expertise:

NA

I confirm that I have read this submission and believe that I have an appropriate level of expertise to confirm that it is of an acceptable scientific standard.

# Reviewer report: Reviewer response for version 1

**DOI:** 10.5256/f1000research.1581.r1023

**Published:** 2013-06-24

**Authors:** Tatsuru Masuda

**Affiliations:** 1 Environmental Sciences, University of Tokyo, Tokyo, Japan

In this paper, the authors have successfully identified a *Chlamydomonas* mutant deficient in chlorophyll biosynthesis using insertional mutagenesis. Through molecular genetic analyses, the authors demonstrated that the deficiency of the *CHLI1* gene, which encodes a subunit of Mg-chelatase, caused the chlorophyll-deficient phenotype. Although *Chlamydomonas* has another homologous *CHLI2* gene, this mutant failed to accumulate chlorophylls, suggesting the limited function of CHLI2 on chlorophyll biosynthesis. To clarify whether the reduced expression of *CHLI2* caused chlorophyll deficiency, overexpression of *CHLI2* in the mutant should give further insights into the functionality of CHLI2. In addition, it is desirable to determine the levels of chlorophyll intermediates that are correlated to the photosensitivity of the mutant. 

**Suggested revisions:**

Considering the distinct regulation of chlorophyll biosynthesis and nuclear gene expression between *Chlamydomonas* and *Arabidopsis*, I am not sure whether this mutant is useful for analysis of the retrograde signaling. Actually, a number of previous papers suggested distinct roles of tetrapyrroles on nuclear gene expression in *Chlamydomonas* ( Kropat *et al*. 1997, Kropat *et al*. 2000, Chekounova *et al.* 2001 and Vos *et al.* 2011). In this sense, the last paragraph of the Discussion section should be largely revised including already known mechanisms in *Chlam ydomonas* cells and not be so focused on *Arabidopsis*.

Reviewer Expertise:

NA

I confirm that I have read this submission and believe that I have an appropriate level of expertise to confirm that it is of an acceptable scientific standard.

# Reviewer report: Reviewer response for version 1

**DOI:** 10.5256/f1000research.1581.r1014

**Published:** 2013-06-20

**Authors:** David Oppenheimer

**Affiliations:** 1 Department of Botany, University of Florida, Gainesville, FL, USA

In this paper, the authors describe the isolation and characterization of a mutant of *Chlamydomonas reinhardtii *deficient in chlorophyll biosynthesis. 

The authors screened a population of Chalamydomonas that was transformed with an insertional mutagenesis vector. They found a chlorophyll deficient mutant that only grew under heterotrophic conditions. The authors provided convincing evidence that this mutant lacked the CHLI1 gene as well as several flanking genes. The *CHLI1* gene encodes a subunit of the enzyme responsible for inserting Mg ^2+^ into protoporphyrin IX. Interestingly, the chli1 mutant also showed decreased expression of a homolog of CHLI1 and CHLI2. The authors also rescued the chli1 mutant phenotype using a wildtype CHLI1 cDNA transgene. 

This work is important because it is the first report of a *chli1* mutant from a green alga. This mutant provides a starting point for additional genetic and biochemical analyses of chlorophyll biosynthesis in *Chlamydomonas*.  

**Suggested revisions:**

The authors should change their mutant name to *chli1-1*, since it is the first mutant allele isolated. This sets a precedent for naming new alleles as they are discovered. 

Instead of calling the rescued transformants that contain the wildtype CHLI1 cDNA "complements", the authors should consider calling them "rescued transformants". Some geneticists adhere to a more strict use of the term "complementation" and its derivatives where complementation is done by crossing two mutants, and not by genetic transformation of a mutant using a wildtype gene.

Reviewer Expertise:

NA

I confirm that I have read this submission and believe that I have an appropriate level of expertise to confirm that it is of an acceptable scientific standard.
